# CGUN-2A: Deep Graph Convolutional Network via Contrastive Learning for Large-Scale Zero-Shot Image Classification

**DOI:** 10.3390/s22249980

**Published:** 2022-12-18

**Authors:** Liangwei Li, Lin Liu, Xiaohui Du, Xiangzhou Wang, Ziruo Zhang, Jing Zhang, Ping Zhang, Juanxiu Liu

**Affiliations:** 1 MOEMIL Laboratory, School of Optoelectronic Science and Engineering, University of Electronic Science and Technology of China, No. 4, Section 2, North Jianshe Road, Chengdu 610054, China; 2 School of Information and Engineering, University of Electronic Science and Technology of China, No. 4, Section 2, North Jianshe Road, Chengdu 610054, China

**Keywords:** machine learning, zero-shot learning, graph convolutional network, contrastive learning, image classification

## Abstract

Taxonomy illustrates that natural creatures can be classified with a hierarchy. The connections between species are explicit and objective and can be organized into a knowledge graph (KG). It is a challenging task to mine features of known categories from KG and to reason on unknown categories. Graph Convolutional Network (GCN) has recently been viewed as a potential approach to zero-shot learning. GCN enables knowledge transfer by sharing the statistical strength of nodes in the graph. More layers of graph convolution are stacked in order to aggregate the hierarchical information in the KG. However, the Laplacian over-smoothing problem will be severe as the number of GCN layers deepens, which leads the features between nodes toward a tendency to be similar and degrade the performance of zero-shot image classification tasks. We consider two parts to mitigate the Laplacian over-smoothing problem, namely reducing the invalid node aggregation and improving the discriminability among nodes in the deep graph network. We propose a top-k graph pooling method based on the self-attention mechanism to control specific node aggregation, and we introduce a dual structural symmetric knowledge graph additionally to enhance the representation of nodes in the latent space. Finally, we apply these new concepts to the recently widely used contrastive learning framework and propose a novel Contrastive Graph U-Net with two Attention-based graph pooling (Att-gPool) layers, CGUN-2A, which explicitly alleviates the Laplacian over-smoothing problem. To evaluate the performance of the method on complex real-world scenes, we test it on the large-scale zero-shot image classification dataset. Extensive experiments show the positive effect of allowing nodes to perform specific aggregation, as well as homogeneous graph comparison, in our deep graph network. We show how it significantly boosts zero-shot image classification performance. The Hit@1 accuracy is 17.5% relatively higher than the baseline model on the ImageNet21K dataset.

## 1. Introduction

Automatic species classification is a problem of great concern in computer vision and pattern recognition. Visual phenomena in practical scenarios generally follow a long-tail distribution [1,2], which leads to a different margin of difficulty in obtaining samples for each category, and some categories do not even have samples. Thus, it is impossible to perform supervised learning on all visual phenomena. Several recent approaches have performed zero-shot learning on some species-specific datasets [3,4]. Reference [3] introduces fusion prototype and hierarchical prototype loss to optimize a network for zero-shot learning on zoological illustrations. Reference [4] proposes a dual autoencoder model for phytoplankton. These existing methods generally use attributes or word vectors, ignoring the explicit hierarchical connections that exist between species. Furthermore, in some emergencies, such as COVID-19 [5,6,7], the ZSL-based model can respond quickly, which improves the accuracy of disease intelligent diagnosis.

In recent years, GCN-based zero-shot learning (ZSL) has shown great potential. Similar to the operation of traditional convolution on Euclidean space, graph convolution enables convolutional operations on the data of non-Euclidean structure by introducing Laplacian matrices as convolutional kernels for weight sharing. This approach allows nodes in the graph to share statistical strength, providing ways for knowledge transfer from the known categories to the unknown in ZSL. Specifically, the GCN-based method is accomplished with both the implicit node feature and explicit hierarchical information. Graph convolution transfers the semantic and structural priors of unlabeled data from source domains, and trains a classifier for classifying unlabeled data in the target domain.

Existing works have pointed out the limitations of graph convolutional neural networks [8,9,10,11]. Multi-layer stacked graph convolutional layers normally raise the Laplacian over-smoothing problem [12,13], which eventually leads the node features in the graph to be similar, thereby reducing the performance of the model. Due to these limitations, most advanced graph convolutional models limit graph convolutional layers to within four layers [11]. Reference [8] gives an insight that a graph convolution is a special form of Laplacian smoothing that aggregates the features of a node and its neighbors to improve the performance on clustering tasks. However, zero-shot classification tasks need to pass messages from known nodes to the unknown in a more fine-grained manner, and the depth of GCN reflects the distance that implicit knowledge can be passed in the graph. Hence, deeper graph convolutional networks enable aggregating features from remote nodes, taking advantage of the global information. Nevertheless, deep graph convolution will exacerbate the effect of smoothing, bringing indistinguishable features between neighboring nodes, which results in a smoothing filter.

To balance the insufficient message passing of shallow graph networks and the severe over-smoothing of deep graph networks, we propose GUN-2A and CGUN-2A. We first propose GUN-2A, a graph convolutional network with five graph convolutional layers. Our intuition is that some nodes after shallow GCN aggregation are analogous to their neighbors and should be discarded before deeper aggregation. We design a novel U-Net-like graph convolutional framework adapted to zero-shot learning. In this method, a new top-k graph pooling scheme based on the self-attention mechanism, Att-gPool, is proposed to highlight under-aggregated nodes. The proposed framework introduces additional implicit knowledge to enhance the representation power of the model without destroying the original topological structure of the knowledge graph. Second, we consider that contrastive learning effectively encourages the intra-class compactness for positive sample pairs and inter-class separability for negative sample pairs. This paradigm seems naturally suitable for node-wise representation in a hybrid knowledge graph and, to some extent, resists the over-smoothing problem. In this case, we propose CGUN-2A. We seamlessly integrate the mentioned graph encoder GUN-2A into a novel graph contrastive learning framework, which utilizes a dual knowledge graph—Hierarchy Knowledge Graph (HKG) and Structural Symmetric Knowledge Graph (SSKG)—to generate visual classifiers for unknown categories via a contrastive loss. The proposed method incorporates various priors for global structural information and fine-grained semantic representation. Experimental results show that our proposed method consistently outperforms existing multi-layer GCN models, which validates the effectiveness of the proposed GUN-2A and contrastive learning modules. Our contributions are as follows:

- We propose a novel graph encoder, GUN-2A, for performing zero-shot image classification individually or applied as an effective graph encoder in the graph contrastive learning module.
- We introduce a Structural Symmetric Knowledge Graph for zero-shot image classification. The additional knowledge graph enhances the representative power of the nodes in the embedding space.
- We propose a graph contrastive learning framework, CGUN-2A. We test it on the most challenging zero-shot image classification dataset, ImageNet-21K, and the result shows that our method significantly outperforms the baseline methods.

## 2. Related Works

### 2.1. ZSL in Ecological Monitoring

With the availability of image sensors, ecologists can easily get access to a large number of ecological pictures or videos based on monitors. However, processing data from surveys remains a major bottleneck in ecology. Deep learning (DL) algorithms have been increasingly used in ecological monitoring. The success of traditional supervised learning methods often depends on a training dataset with a large number of examples. Reference [14] discussed the shortcomings of DL algorithms in ecological monitoring: training bias brought by long-tailed data and misclassification caused by absent data. For the former problem, [15] considered a few-shot learning method based on the feature fusion model and center neighborhood loss for phytoplankton recognition. For the limitation of absent data, [4] proposed a model with a dual-encoder structure for the phytoplankton dataset to perform zero-shot learning. Furthermore, [16] used a ZSL classification framework based on embedding space projection for land cover recognition.

### 2.2. Graph Representation Learning for ZSL and Over-Smoothing Problem

Knowledge transfer is the key to ZSL, which is used to establish relationships between seen and unseen classes. The relationship can be established through implicit semantic information or explicit hierarchical structure. Early works used semantic information, such as manually defined attributes [17,18], word vectors [19,20,21] and textual descriptions [22,23], as source domain. References [24,25,26,27,28,29] attempted to introduce object class hierarchy to constrain the expression of semantic information. Reference [27] developed GCN to encode graph structure and node features according to a first-order approximation of spectral convolutions on graphs. Reference [28] explicitly proposed to learn logistic regression classifiers for unseen classes via GCN. Reference [29] considered the over-smoothing problem of deep GCNs and proposed two shallow networks, SGCN and DGP. DGP exploits the hierarchical structure of knowledge graphs by adding a weighted dense connection scheme.

The over-smoothing problem has been extensively discussed in previous works, mainly concentrating on node classification tasks. DropEdge [30] randomly deletes a certain proportion of edges in the input graph. This method can be regarded as an attenuator for information propagation, generating a sparse variant from the original graph, which avoids the over-smoothing of the deep GCN to a certain extent. Moreover, an important idea to solve the over-smoothing is to expand the range of neighborhood aggregation as much as possible without increasing the depth of the model, such as JK-Net [31], MixHop [32], GDC [33] and APPNP [34]. JK-Net considers the influence of the aggregation radius on information averaging and proposes skip connection, which directly maps the output in the iterative aggregation process to the final output. Similar to dilated convolution, MixHop directly expands the receptive field of the GCN layer by performing mixed feature representations with different hops in the graph, avoiding the use of deep graph networks. GDC uses generalized graph diffusion to eliminate the limitation that the single-layer convolution can only be extended to first-order neighbors. AAPNP separates the neural network that generates predictions from the propagation scheme. In its initial version PPNP, the node attribute is first transformed to obtain an initial node state, and then the personalized PageRank is used to update this state until convergence. However, multiple non-linear operations on the feature matrix in APPNP lead to overfitting. Therefore, APPNP applies a linear combination between different layers; thus, APPNP is still a shallow model, which shows that residual connections are not enough to extend GCN to deep layers [35].

### 2.3. Contrastive Learning for ZSL

Recently, contrastive learning has renewed a surge of interest [36,37,38] in zero-shot learning, including tasks at both the node and graph levels. Several works [39,40] have noted the ability of contrastive learning to mine supervised signals from the data itself, which can be used to address the problem of sparse supervised signals that exist in zero-shot learning. Recent works have successfully applied contrastive learning to zero-shot learning tasks [41,42,43,44]. Reference [41] propose a Transferable Contrastive Network (TCN), which automatically compares images with class semantics to ensure their consistency. Reference [42] aligns triples (state, text, image), in a contrastive manner, in a common embedding space, motivating the model to learn efficient embeddings by exploiting the information in multiple modalities simultaneously. Reference [43] considered the entanglement between state and object, capturing their prototypes in the Siamese contrast embedding space and mitigating the interaction between them. Furthermore, contrastive learning can improve the discriminability between adjacent nodes in the latent embedding space [13,44]. Reference [44] proposed a dual-level contrastive learning network (DCLN) by seamlessly integrating intra-domain and cross-domain contrast learning modules to generate more discriminative features and to ensure explicit knowledge transfer across both domains. The work of [13], which explicitly incorporates a contrastive learning module in a GCN model, is most similar to our research as it also effectively improves the intra-class representation consistency.

## 3. Materials and Methods

### 3.1. Problem Definition

In zero-shot learning settings, assume that set C contains the whole classes, which can be split into the seen classes set $C_{tr}$ and the unseen classes set $C_{te}$. Note that the training set and test set are disjoint, $C_{tr}\cap C_{te}=Ø$, and for each $c_{i}\in C_{tr}$, there is a d-dimensional semantic representation vector $z_{i}\in {\mathbb{R}}^{d}$ corresponding to $c_{i}$. We denote a set of training data point $D_{tr}=\left\{\left(X_{i},c_{i}\right),i=1,\ldots ,M\right\}$, where $X_{i}$ denotes the i-th training image set labeled by $c_{i}$ and M denotes the total number of categories in $C_{tr}$. From then, the common zero-shot learning classification task aims to predict a held-out classifier $\tilde{W}\in {\mathbb{R}}^{N\times d}$ to categorize images of unseen classes based on the training data point, where N contains the number of all categories in both $C_{tr}$ and $C_{te}$.

### 3.2. Preliminary Works

In this section, we summarize some preliminary works, mainly including three parts: Graph Convolutional Learning, Graph U-Nets and Graph Contrastive Learning.

#### 3.2.1. Graph Convolutional Network

Graph convolutional layers are the basis for extensive aggregation in graph networks. We employ the random walk regularization Laplacian matrix [27] as a graph convolution kernel for message propagation and aggregation in the knowledge graph. Specifically, given a knowledge graph G(A,H) containing N nodes, our graph convolution propagation rule is as follows:

(1) $$H^{\left(l+1\right)}=\sigma (D^{-1}\hat{A}H^{\left(l\right)}\Theta^{\left(l\right)})$$

where $H^{\left(l+1\right)}$ is the output feature representation of the l-th GCN layer, and $\Theta^{\left(l\right)}\in {\mathbb{R}}^{d\times F}$ represents the trainable weight matrix of the l-th layer. F is the number of learned convolution kernels. $H^{\left(0\right)}=H$ denotes the input features matrix in the knowledge graph. σ(⋅) denotes the nonlinear activation function LeakyReLU. $D_{ii}=\sum_{j}A_{ij}$ refers to the degree matrix after row regularization to ensure global stability when nodes are aggregated. $\hat{A}=A+I$ represents a degree matrix with self-loops, and I is the identity matrix.

#### 3.2.2. Graph U-Nets

We observed the application of the Graph U-Nets (GUN) proposed in [12], which aims to handle node classification tasks in a semi-supervised way. Consider the great success of U-Nets in pixel-wise prediction tasks that are somewhat similar to zero-shot classification, both of which aim to classify each element in the input. Thus, the U-Net-like structure seems to be well-suited for zero-shot classification tasks. However, it is unnatural to apply this approach to data with graph structure due to the different connectivity between nodes. To bridge the gap, [12], for the first time, presents a novel graph pooling (gPool) and a graph unpooling (gUnpool) operation for top-k selection by linear projection scores. The graph pooling is similar to convolutional pooling, which plays an important role in convolutional neural networks with grid-like data. They can reduce the size of the feature map and enlarge the receptive field, thus improving the generalization performance [45]. Based on these two operations, they propose a U-Net-like architecture for processing graph data.

#### 3.2.3. Graph Contrastive Learning

Most of the previous GCN methods accomplish the aggregation of node features with a single knowledge graph, usually a Hierarchy Knowledge Graph (HKG). HKG is constructed by concepts and hierarchical relationships in WordNet, where concepts are transformed into semantic embeddings by a GloVe text model train on the Wikipedia dataset. Recent developments in contrastive learning have shown the great potential of this approach to regularize consistent representations of different inputs. In particular, this method usually works in a self-supervised manner. The work of [13] introduces contrastive learning into graph representation learning for the first time, aiming at optimizing representations generated by the graph encoder during the feature aggregation phase. The proposed method has illustrated the benefits of hybrid knowledge graphs by additionally constructing explicit semantic relation knowledge graphs for contrastive learning. The contrastive learning module can maximize the agreement of the same nodes in different knowledge graphs whilst expanding the discriminability between different nodes via a contrastive loss in the latent space. This work constructs a heterogeneous K-nearest neighbor graph, Semantic Correlation Knowledge Graph (SCKG), by the top-k sampling of nodes in the original graph. Two simple SGCNs are used to obtain node embeddings on SCKG and HKG, respectively. Note that, here, they perform an end-to-end contrastive learning paradigm, which is different from ours and will be discussed later in Section 3.4.

### 3.3. GUN-2A Architecture

In this section, we will start by describing the framework of GUN-2A, as the improved GUN model fitting for ZSL tasks and the fundamental graph encoder utilized throughout the CGUN-2A in our experiments. Then, we explain the attention-based graph pooling (Att-gPool) layer in detail. Last, the improvement of GUN-2A on GUN will be discussed.

#### 3.3.1. Overview of GUN-2A

**Figure 1:**
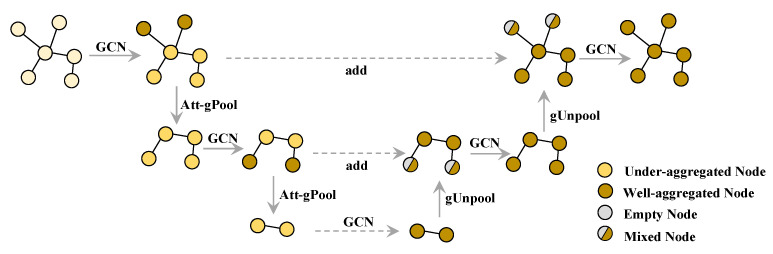
Overview of Graph U-Net with two attention-based graph pooling layers. In this example, the GCN layers aggregate adjacent node features and convert them into a high-dimensional representation. The Att-gPools choose nodes with high attention scores through top-k selection and send them to the next GCN for further aggregation. The gUnpools reconstruct the original graph structure by using position information and empty feature vectors of unselected nodes.

The complete architecture of the proposed encoder, GUN-2A, is shown in Figure 1, which is a key component to accomplishing the transfer of knowledge from semantic space to embedding space. Considering that zero-shot classification on graph data is more sensitive to fine-grained features between nodes, we retain the gUnpool operation in [12] and design a new graph pooling layer based on the self-attention mechanism. We use a Graph U-Net with two attention-based gPool and gUnpool layers as the graph encoder during the training. The encoder aims to output more qualitative representations. We measure the performance of both methods on the ZSL task directly, and the experimental result in Section 4.3 supports this view.

#### 3.3.2. Attention-Based gPool

In this section, we explain our attention-based graph pooling (Att-gPool) layer on graph data. The layer closely follows the work of [12], but the framework adopts a simple projection as its way to calculate the top k score of nodes. We adaptively select a subset of nodes based on the self-attention coefficient to form a new but smaller graph.

Suppose a graph G(A,H) has N nodes, each with d-dimension features, and the relationships between nodes are constructed by experts. Specifically, the graph contains a feature matrix $H\in {\mathbb{R}}^{N\times d}$ and an adjacency matrix $A\in {\mathbb{R}}^{N\times N}$. Each non-zero element in the adjacency matrix A represents an edge between two nodes. Each row vector $h_{i}$ in the feature matrix H refers to the feature vector of a node i.

We take the graph data as input. Given nodes i,j and their feature vector $x_{i,j}$, we compute the attention coefficient $\alpha_{ij}$ on adjacent nodes i,j before the top-k node selection stage.

(2) $$\alpha_{ij}={\mathrm{softmax}}_{j}(e_{ij})=\frac{\exp(\mathrm{LeakyReLU}({\vec{a}}^{T}[W{\vec{h}}_{i}∥W{\vec{h}}_{j}]))}{\sum_{k\in {Ν}_{i}}\exp(\mathrm{LeakyReLU}({\vec{a}}^{T}[W{\vec{h}}_{i}∥W{\vec{h}}_{k}]))}$$

where the weight matrix $W\in {\mathbb{R}}^{d\times d}$ is a learnable linear transformation that transforms the input features into higher-level features. $e_{ij}=a(W{\vec{h}}_{i},W{\vec{h}}_{j})$ represents the importance of the feature of node to node $j\in N_{i}$, and i is a certain neighborhood of node $N_{i}$. In our experiments, we basically follow the setting of [46].

**Figure 2:**
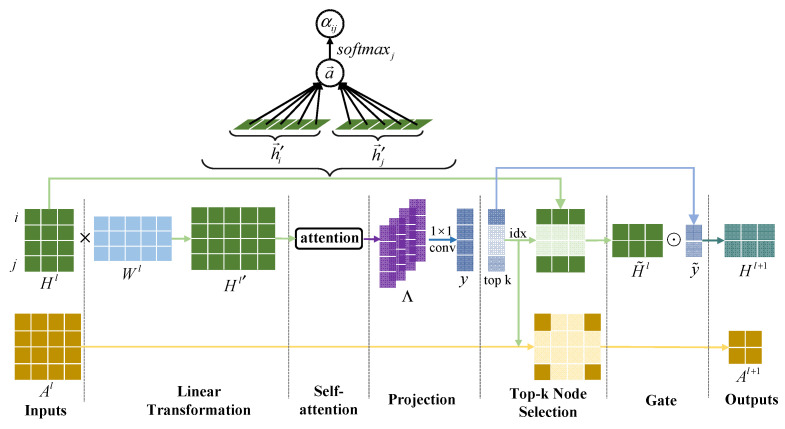
Overview of the proposed attention-based graph pooling layer with k = 2. We take a graph $G(A^{l},H^{l})$ with adjacency matrix $A^{l}\in {\mathbb{R}}^{4\times 4}$ and feature matrix $H^{l}\in {\mathbb{R}}^{4\times 3}$ as input to form a smaller subgraph $G(A^{l+1},H^{l+1})$ with $A^{l}\in {\mathbb{R}}^{2\times 2}$ and $H^{l}\in {\mathbb{R}}^{2\times 3}$. In the linear transformation stage, we use a learnable weight matrix $W^{l}$ to increase the dimension of features and obtain hidden features $H^{l}{}^{\prime }\in {\mathbb{R}}^{4\times 5}$. Then, a shared attentional mechanism is performed to compute attention coefficients. Note that a single-head attention is applied here.$\Lambda \in {\mathbb{R}}^{4\times 4\times 1}$ are the column blocks from the coefficient matrix. The scalar $\alpha_{ij}$ is the projection of the vector ${\vec{h^{\prime }}}_{i}∥{\vec{h^{\prime }}}_{j}$ concatenated by node i,j on the trainable weight vector $\vec{a}$. We use a 1×1 conv to fuse the attention coefficients in node-level and obtain attention score y. Two nodes in $A^{l}$ and $H^{l}$, respectively, with the highest scores are selected in the top-k node selection stage. At the gate stage, we perform element-wise multiplication between ${\tilde{H}}^{l}$ and the selected node scores vector $\tilde{y}$, resulting in $H^{l+1}$.

By calculating the attention coefficients of all nodes, we build the attention coefficient matrix α. Then, we use a 1×1 convolutional layer to build node-wise attention dependencies and perform dimensionality reduction on channels to obtain the top-k score vector y. By top-k sampling the graph, we want to retain as much information as possible from the original graph to fight against over-smoothing. To achieve this, we select the k nodes with the largest attention scores to form a new subgraph. The propagation rule of the Att-gPool layer is shown in Figure 2 and defined as:

(3) $$\begin{array}{l} H^{l}{}^{\prime }=H^{l}\times W,\\ \Lambda =\mathrm{reashpe}\left(\alpha ,(N,N,1)\right),\\ y=\mathrm{conv}\left(\Lambda ,1\right),\\ \mathrm{idx}=\mathrm{rank}\left(y,k\right),\\ A^{l+1}=A^{l}\left[\mathrm{idx},\;\mathrm{idx}\right],\\ H^{l+1}=H^{l}\left[\mathrm{idx},\;:\right]⊙sigmoid{\left(y[idx]\right)}^{T}, \end{array}$$

where $H^{l}$ is the l-th feature matrix as input. Attention coefficient matrix $\alpha \in {\mathbb{R}}^{N\times N}$ is defined by element $\alpha_{ij}$. $\Lambda \in {\mathbb{R}}^{N\times N\times 1}$ represents the column blocks of α. $\mathrm{conv}\left(\Lambda ,1\right)$ denotes the operation of performing 1×1 convolution on Λ. The attention score y measures how much information nodes can retain. idx is returned by $\mathrm{rank}\left(y,k\right)$, which returns indices of the k-largest scores in vector y. $A^{l}\left[\mathrm{idx},\;\mathrm{idx}\right]$ and $H^{l}\left[\mathrm{idx},\;:\right]$ are the row/column extraction to form the adjacency matrix and the feature matrix for the subgraph. $H^{l+1}$ are the network embeddings of the l-th layer, which is used as the input of the next layer. ⊙ means element-wise matrix multiplication.

Differences between GUN-2A and GUN: Compared with the semi-supervised node classification task, the zero-shot learning classification task is more like a regression that needs to output real-valued weights for each node in a fine-grained manner. In this case, the previous GUN may not have enough potential to classify every node in the knowledge graph. Our practice in Section 4.4 illustrates this phenomenon; our re-implementation of GUN has significantly lower performance. We believe that the simple gPool layer in GUN based on computing projection scores does not have sufficient discriminative power to identify similar nodes. Therefore, we reconstruct the whole GUN using the proposed Att-gPool based on self-attention scores. Our goal is to match a model with a U-Net-like structure to the zero-shot learning classification task. The GUN-2A samples the subset of important nodes to make sure that highly abstract features are encoded and the receptive field is enlarged. In addition, to transfer such a model to ZSL, we have to make some compromises, such as the $k^{th}$ graph power being removed and the feature dimension increasing from 300 to 2049. More details of re-implementation are described in Section 4.2.

### 3.4. CGUN-2A Architecture

In this section, to begin with, we present the overall framework of CGUN-2A. Next in importance, the Structural Symmetric Knowledge Graph is described for maintaining the dual semantic knowledge for contrastive learning. Once again, we explain how the CGUN-2A is used for ZSL. In the end, differences between DKG [13] and CGUN-2A are also discussed.

#### 3.4.1. Overview of CGUN-2A

**Figure 3:**
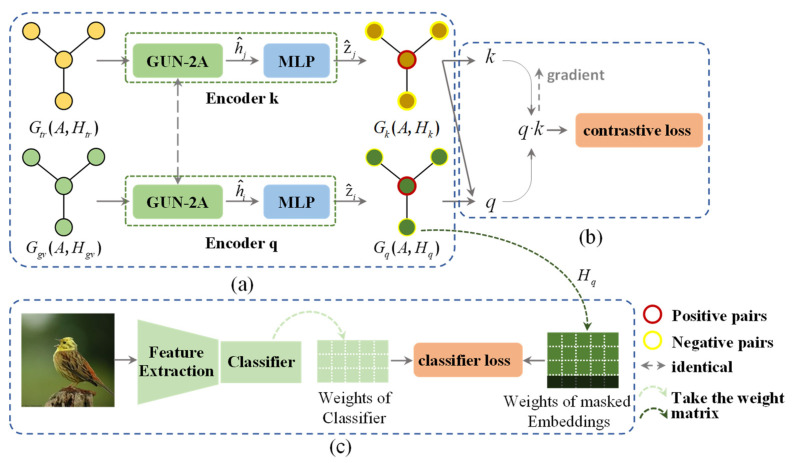
The overall framework of the proposed zero-shot classification model CGUN-2A. The model is constructed by (**a**) graph encoder, (**b**) contrastive learning module and (**c**) classifier learning module.

Besides the widely used GolVe-encoded knowledge graph $G_{gv}$, we additionally use the semantic encoder in CLIP Transformer [47] with the WordNet hierarchy [48] to build a dual homogeneous knowledge graph, $G_{tr}$, in another semantic space. This dual graph is taken as the input of the model. The model consists of three parts: graph encoder, contrastive learning module and classifier learning module, as shown in Figure 3. The graph encoder contains a GUN-2A and an MLP component. During encoder updating, we map the dual graph to the embedding space H with two invariant spread GUN-2A, respectively. Then, we use two MLPs to output the embeddings separately to the latent common space Z. In the contrastive learning phase, we define the proxy task in a common space and maximize the gap between positive and negative samples by contrastive loss. Finally, the model is trained to predict the classifier weights for the unseen classes by optimizing the classifier loss between the last layer weights of a pre-trained visual feature extractor and the seen node embeddings in common space.

#### 3.4.2. Structural Symmetric Knowledge Graph

**Figure 4:**
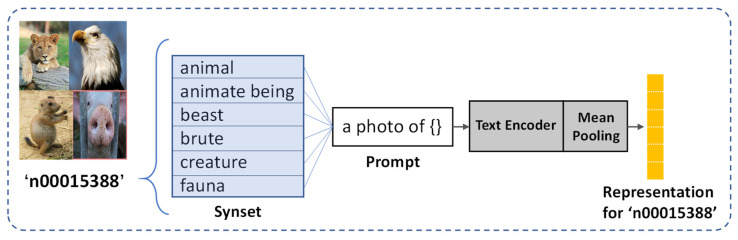
An illustration for generating the representation of class ‘n00015338′ in ImageNet21K, which is a wnid concept consisting of 6 synonyms.

In addition to the widely spread hierarchy knowledge graph, we further explore the potential of CLIP [47] used in the construction knowledge graph. CLIP has now become a de facto general-purpose framework for visual-semantic tasks because it establishes a strong connection between visual and semantic signals, and is also very convenient for downstream tasks. For this purpose, we use the CLIP Transformer text encoder to obtain a class representation for each concept. We first pick up the WordNet id (wnid) of each concept and get all the words in the synset of the wnid. Note that about half of the class concepts in ImageNet21K contain more than one synonym. Then, prompt engineering is used to create a prompt template for each word, and we take it as input for the CLIP text encoder. Finally, we perform mean pooling on all synonym semantic vectors and obtain the final class representation of the concept. A specific example is shown in Figure 4.

#### 3.4.3. CGUN-2A for ZSL

In the graph contrastive learning module, the inputs of our module are two topological correlation knowledge graphs,$G_{gv}(A,H_{gv})$ and $G_{tr}(A,H_{tr})$, as shown in Figure 3b, which are encoded by the pre-trained semantic encoders Glove and CLIP Transformer, respectively. Note that the dual knowledge graph has a consistent topology, built by the WordNet hierarchy. We take the feature $h_{i}^{gv}$ of node i in the knowledge graph $G_{gv}$ as the input $x^{query}$ of the encoder q. The feature $\left\{h_{i}^{tr},h_{j}^{tr},j=1,2,\ldots \right\}$ of the corresponding node i and its neighbor nodes $j\in N_{i}$ in the knowledge graph $G_{tr}$ is used as the input $x^{key}=\left\{x_{0}^{key},x_{1}^{key},\ldots \right\}$ of the encoder k. Encoders q and k are two invariant spread GUNs. We define the proxy task as a pair of positive samples formed by the feature $h_{i}^{gv},h_{i}^{tr}$ of the adjoint node i in $G_{gv},G_{tr}$, respectively, and the feature $h_{j}^{gv,tr}$ of node $j\in N_{i}$ as a negative sample of $h_{i}^{gv}$. In our experiments, we randomly sample K nodes from the second-order neighborhood of node i as negative samples. In addition, following the observation of [49], we introduce a learnable nonlinear transformation before the contrastive loss to obtain sufficient expressive power. To that end, we use an MLP with one hidden layer to map the representation ${\hat{h}}_{i}$ to the space of contrast loss effects, $q_{i}=W^{\left(2\right)}\sigma \left(W^{\left(1\right)}{\hat{h}}_{i}\right)$, where σ is the ReLU nonlinear activation function. For the output encoded query q, there exists a set of encoded samples $\left\{k_{+},k_{j},j=1,2,\ldots \right\}$. Note that there is a single key (denoted as $k_{+}$) in the dictionary that q matches. Our goal at this stage is to use a contrastive loss to maximize the difference between positive samples $k_{+}$ and negative samples $k_{j}$ by sampling in different signal spaces to alleviate Laplacian over-smoothing. With similarity measured by dot product, the loss function of contrastive learning [50] is formulated as

(4) $$L_{q}=-\log\frac{\exp(q\cdot k_{+}/\tau )}{\sum_{j=0}^{K}\exp(q\cdot k_{j}/\tau )}$$

where τ is a temperature hyper-parameter [51] that controls the shape of the logits’ distribution.

Our model builds upon the Contrastive Graph U-Net. Inspired by [28,29], the purpose is to output a classifier for each unseen class. In the inference stage, we use this classifier directly as head for visual features to preform zero-shot classification, as shown in Figure 3. As an initial step, we use the semantic hidden states $H_{q}$ output by CGUN-2A to initialize the predicted classifier weights ${\tilde{W}}^{N}\in {\mathbb{R}}^{N\times d}$. The final predicted visual classifier is obtained by optimizing masked L2 Loss [29]:

(5) $${\text{ℒ}}_{classifier}=\frac{1}{2M}{\sum_{i=1}^{M}\sum_{j=1}^{d}\left(W_{i,j}^{M}-{\tilde{W}}_{i,j}^{M}\right)}^{2}$$

where $W^{M}\in {\mathbb{R}}^{M\times d}$ denotes the ground truth weights for seen categories obtained by extracting the last layer weights of a pre-trained CNN. Note that we mask the last N−M unseen categories in $W_{i,j}^{N}$, and only use the first M seen categories. Finally, our total loss includes the loss from the contrastive learning and classifier learning stages and is defined as:

(6) $${\text{ℒ}}_{all}={\text{ℒ}}_{contrast}+\lambda {\text{ℒ}}_{classifier}$$

where λ is the weight parameter.

During the inference phase, we use the obtained classifier to replace the last layer of the pretrained CNN model for prediction.

**Figure 5:**
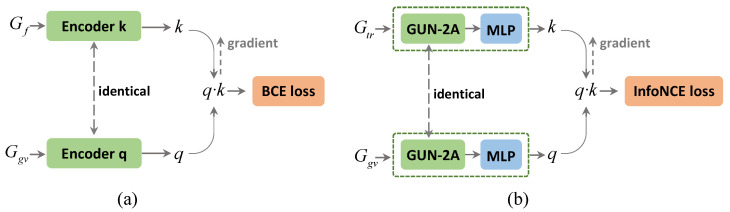
A paradigm comparison of the contrastive learning module in DKG (**a**) and CGUN-2A (**b**) with different inputs, graph encoders and loss function.

Differences between CGUN-2A and DKG: The main distinctions are summarized in three aspects: knowledge graphs as input, graph encoder and loss function. First, we apparently use different dual knowledge graphs. DKG seems to use an HKG and a skip-connected HKG, which can be thought of as a contrast between SGCN and DGP. In our dual knowledge graph, we preserve the original structure of the graph while introducing additional semantic information in a loose coupling manner. Second, based on the proposed graph aggregator GUN-2A, we construct a graph encoder with higher efficiency, which makes full use of the hierarchical information in the knowledge graph. Last, in our task, we want to generate stronger intra-class compactness and inter-class separability for the node representations of the two outputs, q and k. We take the InfoNCE loss, which is designed to have a low value when the query is similar to its positive key and dissimilar to all other keys. We draw a simple comparison of the two modules we mentioned in Figure 5.

## 4. Discussion of Results

### 4.1. Experimental Settings

We performed a comparative evaluation of our experiments on ImageNet [52], currently the large-scale and most widely used dataset for zero-shot learning. The categories in ImageNet are constructed based on the WordNet hierarchy, based on which we can organize the categories in the form of a knowledge graph. Reference [19] proposed to use the ImageNet 21K dataset for zero-shot learning. Our work follows the splitting setting of the training/test set of the previous work [13,19,28,29,53]. Specifically, we take the 1000 categories in the ISLVRC 2012 dataset with WordNet hierarchy as the seen classes, and the 20842 categories in the ImageNet 21K dataset as the unseen class set. Note that images in the unseen dataset are completely invisible during the training. ImageNet 21K is divided into three benchmark datasets of increasing difficulty: “2-hops”, “3-hops” and “all”. Furthermore, we use the same metric, Hit@k, which represents the percentage of the top-k predictions that hit ground truth labels. We compare our CGUN-2A to the following methods, EXEM [19], GCNZ [28], SGCN [29], DGP [29], DKG [13] and ZSL-KG [53].

### 4.2. Implementation Details

Consistent with previous works [13,28,29], we adopt the ResNet-50 [54] model pretrained on the ISLVRC 2012 dataset as the visual feature extractor. In the contrastive learning phase, we use two text feature extractors, CLIP Transformer [47] and GloVe [55] trained on the Wikipedia dataset. CGUN-2A uses two attention-based graph pooling layers and two graph unpooling layers. The downsampling rates of the encoder GUN-2A are set to 0.7 and 0.5. The number of randomly sampled nodes in the contrastive learning module is K=20, and the shortages are supplemented by orthogonal vectors of the sampled node if the node does not have enough neighbors. Similarly, we perform Dropout with a dropout rate of 0.5 in each layer. Our model is trained with 300 epochs, using Adam with a learning rate of 0.001 and a weight decay of 0.0005. The proposed CGUN-2A model is trained on a GTX 3090 GPU.

### 4.3. Performance Comparison

The results of our comparative evaluation experiments on three datasets are summarized in Table 1. The proposed method CGUN-2A significantly outperforms previous works on three benchmark datasets, including EXEM [19], GCNZ [28], SGCN [29], DGP [29], DKG [13] and ZSL-KG [53]. Furthermore, we implement Transformer-encoded knowledge graphs in SGCN and DGP on the “2-hops” dataset and achieve some performance improvements. Specifically, compared to the “2-hops” and “3-hops” datasets, our method outperforms the previous models on the “All” task with a considerable margin, achieving a relative improvement of 17.5% in Top-1 accuracy, illustrating that the proposed method works at the global level of the graph and effectively resists over-smoothing. We also test the performance of the encoder GUN-2A in our model separately on the zero-shot classification task, and the results outperform the shallow SGCN and GCNZ with one and four layers on all three datasets. In particular, GUN-2A outperforms DGP on the “3-hops” and “all” datasets, illustrating the effectiveness of our encoder in aggregating global knowledge.

In the test involving seen classes, namely generalized zero-shot learning (GZSL), we add the weights of both seen and unseen classes to the final predictive classifier, and the results are shown in Table 2. Compared with Table 1, we observe that the accuracy is considerably lower, but the CGCN-2A has a relative improvement of 40% in top-1 accuracy in the “all” dataset compared to previous methods. Compared with the relative improvement of 17.5% in Table 1, we believe that although the smoothing exists, its impact is greatly reduced. Moreover, CGUN-2A still outperforms the previous state-of-the-art method DKG by a large margin on all tasks for low k in the Top-k accuracy measure. We confirm that the proposed encoder structure and the introduced structural symmetric knowledge graph make more efficient use of implicit knowledge.

### 4.4. Analysis of Smoothness

We quantify our two proposed frameworks based on the mean average distance (MAD) [9] metric. MAD measures the smoothness (cosine distance) of output representations by computing the mean average distance among the predicted weights of the visual classifier. We performed calculations using both GZSL and CZSL settings on the “2-hops”, “3-hops, and “all” benchmark datasets, and the results are shown in Table 3. We observe the effectiveness of our method in promoting the inter-class separability of prediction weights. Note that our method is a model with five GCN layers, while both SGCN and DGP are shallow models. From the observation in [9], as the number of model layers increases, the smoothness of the graph representation also rises significantly. Additionally, in the GZSL settings, we can observe an overall increase in the smoothness of the output results, which verifies that the mentioned over-smoothing is the real problem where the GCN-based methods are difficult to work in the GZSL settings.

### 4.5. Analysis of Ablation

We conduct ablation studies to verify the effectiveness of each component in our proposed method. As is shown in Table 3, we implement some variants of the proposed model and report the comparison results. From Table 4, we note the following. (1) the model using the Transformer-encoded knowledge graphs $G_{tr}$ performs better than GloVe-encoded knowledge graph models. (2) The model that integrates both $G_{tr}$ and $G_{tr}$ achieves better performance than the model with either $G_{tr}$ or $G_{tr}$ only. This shows that combining both $G_{tr}$ and $G_{tr}$ can capture more complete correlation information among categories. (3) The performance of our model is better than other variants. This demonstrates the capability of our graph contrastive learning module to generate discriminative and effective classifiers.

### 4.6. Analysis of the Number of Layers

We perform an empirical evaluation to verify that our intuition is correct and that additional hidden layers in GUN-2A indeed aggregate more information from the global structure in the knowledge graph. Table 5 reports the model performance of previous similar works [28,29] using GCN with a different number of layers and illustrates the performance when adding additional layers to the GUN for 2-hops task. We observe a steady decline in the performance of previous methods as the number of layers increases. In our experiments, the performance of our model increases with more GCN layers. Compared to the 4-layers GCNZ, we believe that our GUN structure effectively aggregates valid features from remote nodes without modifying the origin topology. However, this aggregation also brings potential concerns of gradients vanishing as the number of GCN layers continues to increase.

## 5. Conclusions

In this paper, we propose a new zero-shot image classification method in ecological monitoring. The proposed method explicitly exploits the hierarchy among natural creatures and explores multiple relations between different classes for learning the weight of a visual classifier. The proposed encoder GUN-2A achieves broad cross-node knowledge propagation. The introduced structural, symmetric knowledge graph and graph contrastive learning module substantially alleviate the Laplacian over-smoothing problem in a single knowledge graph and enhances the distinguishability of the predicted classifier. To validate the potential of our method in real-world environments, we tested it on several large-scale benchmark datasets. Our experimental results demonstrate the effectiveness of the proposed zero-shot learning framework.

## Figures and Tables

**Table 1:** Hit@k performance for different methods on three datasets. Only testing on the unseen classes.

Test Set	Num of Class	Model	Layer of GCN	Hit@k (%)				
				1	2	5	10	20
2-hops	1549	EXEM	-	12.5	19.5	32.3	43.7	55.2
		GCNZ	4	19.8	33.3	53.2	65.4	74.6
		SGCN	1	26.2	40.4	60.2	71.9	81.0
		DGP	1	26.6	40.7	60.3	72.3	81.3
		SGCN(Tr) ^1^	1	28.2	43.3	62.7	74.1	82.3
		DGP(Tr) ^1^	1	27.0	41.4	61.3	73.0	81.5
		DKG	1	28.4	43.0	62.6	74.5	82.9
		ZSL-KG(Tr) ^1^	1	26.6	40.7	60.3	72.3	81.3
		GUN-2A(ours)	5	26.4	40.5	60.3	71.8	80.6
		CGUN-2A(ours)	5	**29.2**	**43.6**	**63.0**	**74.6**	**82.9**
3-hops	7860	EXEM	-	3.6	5.9	10.7	16.1	23.1
		GCNZ	4	4.1	7.5	14.2	20.2	27.7
		SGCN	1	6.0	10.4	18.9	27.2	36.9
		DGP	1	6.3	10.7	19.3	27.7	37.7
		DKG	1	7.0	11.7	20.7	29.2	39.0
		ZSL-KG(Tr) ^1^	1	6.3	11.1	20.1	28.8	38.8
		GUN-2A(ours)	5	6.4	10.8	19.5	27.9	38.0
		CGUN-2A(ours)	5	**7.8**	**13.0**	**22.6**	**31.5**	**41.8**
All	20842	EXEM	-	1.8	2.9	5.3	8.2	12.2
		GCNZ	4	1.8	3.3	6.3	9.1	12.7
		SGCN	1	2.8	4.9	9.1	13.5	19.3
		DGP	1	3.0	5.0	9.3	13.9	19.8
		DKG	1	3.3	5.6	10.1	14.7	20.5
		ZSL-KG(Tr) ^1^	1	3.0	5.3	9.9	14.8	21.0
		GUN-2A(ours)	5	3.0	5.3	9.7	14.4	20.3
		CGUN-2A(ours)	5	**4.0**	**6.7**	**12.2**	**17.8**	**24.8**

^1^ Tr means Transformer-encoded.

**Table 2:** Hit@k performance for different methods on three datasets. Testing on both the seen and unseen classes.

Test Set	Num of Test Class	Model	Hit@k (%)				
			1	2	5	10	20
2-hops + 1K	1549	GCNZ	9.7	20.4	42.6	57.0	68.2
		SGCN	11.9	27.0	50.8	65.1	75.9
		DGP	10.3	26.4	50.3	65.2	76.0
		DKG	7.0	26.8	52.5	67.5	77.9
		ZSL-KG(Tr) ^1^	11.1	26.2	50.0	64.3	75.3
		GUN-2A(ours)	11.2	26.8	50.4	65.2	75.4
		CGUN-2A(ours)	**13.5**	**28.9**	**52.9**	**65.9**	**76.3**
3-hops + 1K	7860	GCNZ	2.2	5.1	11.9	18.0	25.6
		SGCN	3.2	7.1	16.1	24.6	34.6
		DGP	2.9	7.1	16.1	24.9	35.1
		DKG	2.0	7.1	17.3	26.2	36.5
		ZSL-KG(Tr)^1^	3.4	7.5	16.9	26.1	36.5
		GUN-2A(ours)	2.9	6.9	16.0	24.7	34.3
		CGUN-2A(ours)	**4.6**	**9.4**	**19.6**	**29.0**	**39.6**
All + 1K	20842	GCNZ	1.0	2.3	5.3	8.1	11.7
		SGCN	1.5	3.4	7.8	12.3	18.2
		DGP	1.4	3.4	7.9	12.6	18.7
		DKG	1.0	3.4	8.5	13.2	19.3
		ZSL-KG(Tr)^1^	1.7	3.8	8.5	13.5	19.9
		GUN-2A(ours)	1.4	3.4	8.6	12.7	18.3
		CGUN-2A(ours)	**2.5**	**5.1**	**10.8**	**16.5**	**23.7**

^1^ Tr means Transformer-encoded.

**Table 3:** The MAD values of some GCN-based methods on the ‘2-hops’, ‘3-hops’ and ‘all’ datasets with both CZSL and GZSL settings.

Model	MAD for CZSL			MAD for GZSL		
	2-Hops	3-Hops	All	2-Hops	3-Hops	All
SGCN	0.431	0.389	0.411	0.258	0.344	0.386
DGP	0.375	0.367	0.387	0.228	0.327	0.378
GUN-2A	0.429	0.391	0.418	0.265	0.345	0.377
CGUN-2A	0.453	0.419	0.433	0.275	0.372	0.413

**Table 4:** Results for the ablation study for 2-hops. $G_{gv}$ and $G_{tr}$ represent graphs encoded by the encoders Glove and Transformer, respectively.

Model	$G_{gv}$	$G_{tr}$	Att-gPool	Hit@k (%)				
				1	2	5	10	20
GUN	✓	✗	✗	17.4	28.0	45.0	58.9	71.2
GUN-2A	✓	✗	✓	26.4	40.5	60.3	71.8	80.6
	✗	✓	✓	28.4	42.5	61.5	72.9	81.3
CGUN-2A	✓	✓	✓	29.2	43.6	63.0	74.6	82.9

**Table 5:** Results for 2-hops for the model with different depths.

Model	GCN Layer	Hit@k (%)				
		1	2	5	10	20
SGCN *	1	24.8	38.3	57.5	69.9	79.6
	2	24.2	37.7	57.4	69.2	78.1
	3	23.9	37.5	57.1	68.4	77.2
GCNZ	4	19.8	33.3	53.2	65.4	74.6
GUN-None	2	24.2	37.6	57.1	69.6	79.4
GUN-1A	3	25.4	39.0	58.5	70.3	79.5
GUN-2A	5	**26.4**	**40.5**	**60.3**	**71.8**	**80.6**
GUN-3A	7	25.5	39.3	58.6	71.1	79.4

* indicate variants of SGCN without finetuning.

## Data Availability

The algorithm codes and our dataset will be released online at www.github.com/lil-wayne-0319/CGUN-2A (accessed on 14 December 2022).
